# Nitrogen and Phosphorus Signaling and Transport During Legume–Rhizobium Symbiosis

**DOI:** 10.3389/fpls.2021.683601

**Published:** 2021-06-22

**Authors:** Yanlin Ma, Rujin Chen

**Affiliations:** ^1^MOE Key Laboratory of Cell Activities and Stress Adaptations, Lanzhou University, Lanzhou, China; ^2^School of Life Sciences, Lanzhou University, Lanzhou, China

**Keywords:** legume–rhizobium symbiosis, nitrogen fixation, nitrogen and phosphorus signaling, transport, crosstalk

## Abstract

Nitrogen (N) and phosphorus (P) are the two predominant mineral elements, which are not only essential for plant growth and development in general but also play a key role in symbiotic N fixation in legumes. Legume plants have evolved complex signaling networks to respond to both external and internal levels of these macronutrients to optimize symbiotic N fixation in nodules. Inorganic phosphate (Pi) and nitrate (NO_3_^−^) are the two major forms of P and N elements utilized by plants, respectively. Pi starvation and NO_3_^−^ application both reduce symbiotic N fixation *via* similar changes in the nodule gene expression and invoke local and long-distance, systemic responses, of which N-compound feedback regulation of rhizobial nitrogenase activity appears to operate under both conditions. Most of the N and P signaling and transport processes have been investigated in model organisms, such as *Medicago truncatula*, *Lotus japonicus*, *Glycine max*, *Phaseolus vulgaris*, *Arabidopsis thaliana*, *Oryza sativa*, etc. We attempted to discuss some of these processes wherever appropriate, to serve as references for a better understanding of the N and P signaling and transport during symbiosis.

## Introduction

Legume plants can establish symbiotic interactions with soil bacteria, collectively known as rhizobia, to form new organs, i.e., nodules, in which differentiated bacteroids enclosed in plant cell-derived membranes in nodule-infected cells reduce atmospheric dinitrogen (N_2_) to ammonium (NH_3_) as a nitrogen (N) source for the growth of the host. The establishment of symbiotic interactions involves extensive dialogs of the signaling processes between the plant host and bacterial partners (Oldroyd and Downie, 2008; Oldroyd et al., 2011; Oldroyd, 2013).

The nitrogenase complex of bacteroids in nodules catalyzes the reduction of N_2_ to NH_3_. This process is energy-intensive, which requires a significant input of photosynthates (approximately equivalent to 25% of the shoot dry matter at harvest) from the host (Schulze et al., 1999). To minimize the energy cost, legume plants preferentially use any alternative N sources for growth, and when alternative N sources are not available or environmental conditions are unfavorable for growth, they restrict the nodule N_2_ fixation to the minimal level necessary to support plant growth. For optimal growth, legume plants have evolved a long-distance (systemic) regulatory mechanism [termed as autoregulation of nodulation (AON)] to control the number of nodules formed on the roots of a plant, and therefore the total nitrogenase activity and mechanisms quickly and efficiently inhibit the N fixation activity of nodules when alternative N sources are available or under environmental stresses (Magori and Kawaguchi, 2009; Mortier et al., 2012; Cui et al., 2019; Kobayashi et al., 2020).

Phosphorus (P) is an essential macronutrient required for the biosynthesis of nucleic acids, phospholipids, and many other molecules and metabolites in plants (Richardson, 2009). Not surprisingly, a deficiency in inorganic phosphate (Pi) severely affects photosynthesis, photorespiration, and other essential processes, and thus reducing plant growth. A sufficient supply of Pi is also required for nodule development and symbiotic N fixation in legumes (Gunawardeba et al., 1992; Vance et al., 2003; Sulieman and Tran, 2015). Pi is largely immobile and has poor mobility in soil, and therefore it is often not easily accessible by plant roots (Holford, 1997). As a result of adaptation, plants have evolved various mechanisms to cope with growth under the condition of low Pi. These include morphological and growth changes, changes in the transcription of a large number of Pi starvation-responsive genes encoding transcription factors, Pi transporters, and scavengers. In legume plants, Pi starvation and nitrate (NO_3_^−^) application both inhibit symbiotic N fixation. In this review, we first provide a brief overview of a transcription factor NODULE INCEPTION (NIN) and its involvement in nodulation, followed by an overview of the role of NIN-like transcription factors in the NO_3_^−^ inhibition of nodule development, NO_3_^−^ and NH_4_^+^ transporters, nitric oxide (NO), and Pi signaling and transport in symbiotic N fixation. Finally, we provide a brief overview of the transcriptional regulation of Pi starvation and NO_3_^−^ signaling.

## Nodule Inception, a Key Transcription Factor For Regulating Rhizobial Infection and Nodule Organogenesis

**Figure 1:**
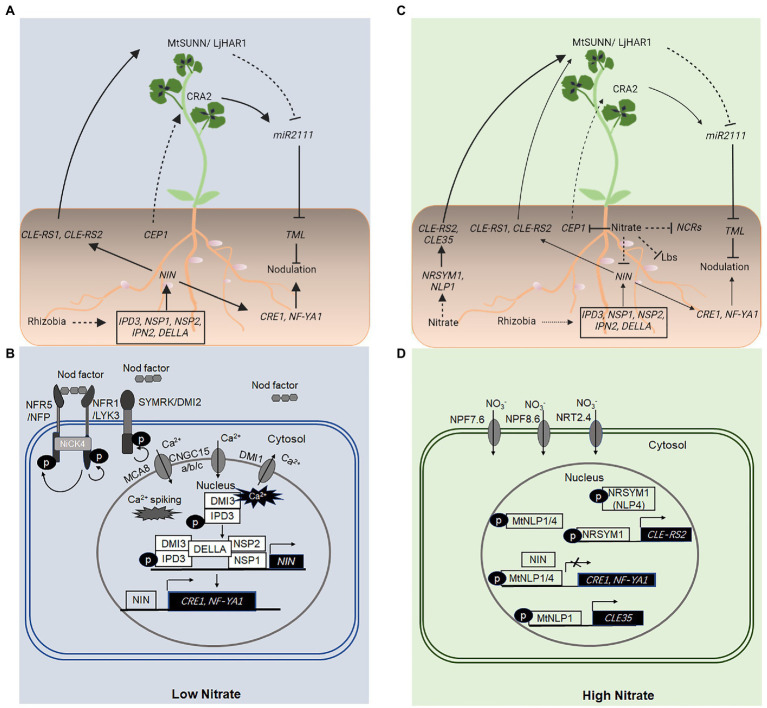
Local and long-distance, systemic regulation of nodulation using NODULE INCEPTION (NIN) and NIN-like proteins (NLPs). **(A,B)** Under conditions of low nitrate (NO_3_^−^), *NIN* activation is downstream from the initial perception of rhizobial nod factors by the LysM receptor-like kinases NFR1 and NFR5 in *Lotus japonicus* and LYK3 and NFP in *Medicago truncatula*, localized in the plasma membrane of root epidermal cells. Nodulation factor (NF) perception and signal transduction also require the LRR-RLK, SYMRK/DMI2, a cytoplasmic receptor-like kinase, NFR5-INTERACTING CYTOPLASMIC FORWARDING4 (NICK4), and a perinuclear Ca^2+^ oscillation (Ca^2+^ spiking) mediated by several proteins, including DOES NOT MAKE INFECTION1 (DMI1), CNGC15a/b/c, and MCA8 located in distinct nuclear membranes. The Ca^2+^ oscillation is perceived by the CALCIUM/CALMODULIN-DEPENDENT PROTEIN KINASE (CCaMK), DMI3, which interacts with and phosphorylates the transcription factor CYCLOP/IPD3. DELLA proteins promote DMI3–IPD3 interactions and phosphorylation of IPD3, and interactions between IPD3–NSP2–NSP1 complexes, which bind distinct *cis*-elements in the promoter sequence of *NIN* and activate *NIN* expression. The NF-induced NIN activation upregulates the expression of downstream genes including *NF-YA1* and *CRE1*, which are essential for rhizobial infection and nodule organogenesis, respectively. NIN also binds directly to the promoter sequence and activates the expression of genes encoding CLE-like peptides such as CLE-RS1 and CLE-RS2 in *L. japonicus*, which are transported from the root to shoot, where they are perceived by the LRR-RLK, LjHAR1/MtSUNN. This leads to a reduced level of the mobile signal, *miR2111* in the shoot and the root, and a corresponding increase in the transcript level of the *miR2111* target, *TML*, which encodes a negative regulator of nodule initiation in the root. Conditions of low nitrogen (N) also activate a CEP1-CRA2 systemic pathway, which positively regulates the nodule number. **(C,D)** A high level of NO_3_^−^ inhibits nodulation both locally and systemically. A high level of NO_3_^−^ activates the expression of genes encoding the NLPs, NLP1, and *NITRATE UNRESPONSIVE SYMBIOSIS1* (*NRSYM1*; *NLP4*), which directly activate the expression of genes encoding CLE35 and CLE-RS2 peptides in *M. truncatula* and *L. japonicus*, respectively. CLE-RS2 and CLE35 peptides function to inhibit the number of nodules formed on roots *via* the LjHAR1/MtSUNN-dependent autoregulation of nodulation (AON) pathway. A high level of NO_3_^−^ also suppresses the *CEP1* gene expression, diminishing the activity of a CEP1-CRA2 systemic pathway in the nodule number regulation. **(D)** Under conditions of high NO_3_^−^, NLPs (NLP1–NLP4 in *M. truncatula*) are activated and accumulate in the nucleus, where they interact with NIN and compete with NIN for binding sites in promoter sequences of target genes or both. As a result, the expression of NIN-activated genes including *CRE1* and *NF-YA1* is suppressed, inhibiting nodulation. A high level of NO_3_^−^ also suppresses the expression of nodule cysteine-richs (*NCRs*) and *LEGHEMOGLOBIN (Lbs)*, which results in the inhibition of nitrogenase activity. Solid and dashed lines indicate the processes that are supported by experimental evidence and putative, respectively. The size of the arrows indicates the relative strength of the signaling process.

Nodule development involves two separate processes, rhizobial infection, which takes place in root epidermal cells, and nodule organogenesis that is initiated in root cortical cells (Oldroyd and Downie, 2008). The nodule-specific transcription factor NIN plays an essential role in both processes (Schauser et al., 1999; Marsh et al., 2007). Under conditions of low soil N, *NIN* activation is downstream from the initial perception of rhizobial Nod factors [Nodulation factors (NFs)] by the plasma membrane-localized LysM domain receptor-like kinases NFR1 and NFR5 in *Lotus japonicus* and LYK3 and NFP in *Medicago truncatula* (Limpens et al., 2003; Madsen et al., 2003; Radutoiu et al., 2003; Indrasumunar et al., 2010; Ried et al., 2014; Figure 1). The leucine-rich repeat receptor-like kinase SYMRK/DMI2 (*L. japonicus*/*M. truncatula* counterparts) also plays a key role in this process (Stracke et al., 2002; Limpens et al., 2005). The receptor-like cytoplasmic kinase NFR5-INTERACTING CYTOPLASMIC FORWARDING4 (NICK4) may link the NF signaling to downstream processes (Wong et al., 2019; Figure 1).

Downstream from the NF signaling is a perinuclear Ca^2+^ oscillation (or calcium spiking) in root epidermal cells, which requires several proteins, including CYCLIC NUCLEOTIDE-GATED CHANNELs15a/b/c (CNGC15a/b/c), *M. truncatula* SERCA-Type Ca^2+^-ATPase8 (MtMCA8), and DOES NOT MAKE INFECTION1 (DMI1) located in distinct nuclear membranes (Capoen et al., 2011; Venkateshwaran et al., 2012; Charpentier et al., 2013, 2016; Leitao et al., 2019; Figure 1). The NF-induced perinuclear Ca^2+^ oscillation is perceived by the CALCIUM/CALMODULIN-DEPENDENT PROTEIN KINASE (CCaMK), DMI3 (Messinese et al., 2007), which specifically interacts with and phosphorylates the transcription factor CYCLOPS (also known as IPD3) in *M. truncatula* (Messinese et al., 2007; Yano et al., 2008). The phosphorylated IPD3 activates the transcription of *NIN* (Marsh et al., 2007; Messinese et al., 2007), which in turn upregulates the expression of downstream genes, including, among others, *NF-YA1* and *CRE1* required for rhizobial infection and nodule organogenesis, respectively (Vernie et al., 2015; Lin et al., 2018; Figure 1).

Notably, MtDELLA proteins (MtDELLA1, 2, 3), members of the plant-specific GRAS [GIBBERELLIN-INSENSITIVE (GAI), REPRESSOR OF *ga1-3* (RGA) and SCARECROW (SCR)] protein family and key negative regulators of gibberellin signaling, promote the interaction between DMI3 (CCaMK) and CYCLOP/IPD3 and the phosphorylation of IPD3 (Jin et al., 2016). MtDELLA proteins also promote the interactions between IPD3 and NODULATION SIGNALING PATHWAY2 (NSP2), a different member of the GRAS family (Jin et al., 2016). Phosphorylated CYCLOP/IPD3 and NSP1-NSP2 complex can bind a CYC-box (TGCCATGTGGCA) and an NRE-box (AATTT) in the promoter sequence and activate the expression of *NIN* (Jin et al., 2016; Figure 1).

## Nitrate Inhibition of Nodulation Mediated By Nin-Like Transcription Factors

NODULE INCEPTION belongs to a NIN-like protein (NLP) subfamily of the plant-specific RWP-RK family of transcription factors (Wu et al., 2020). An NLP subfamily differs from the RWP-RK domain (RKD) subfamily in which, in addition to the RKD, they also contain in their C-terminal, the Phox and Bem1 (PB1) domain, which is involved in protein–protein interactions with other PB1 domain proteins. Although the functions of most RWP-RK proteins remain unknown, recent studies have shown that NLPs play an important role in NO_3_^−^ signaling, and response in *Arabidopsis* and several other plant species (Mu and Luo, 2019). Lin et al. (2018) have identified five *NLP* genes in *M. truncatula* (Lin et al., 2018). *MtNLP1–4* genes exhibit tissue-specific expression patterns, albeit most of them express in roots and nodules (Lin et al., 2018). Interestingly, *M. truncatula* transgenic hairy roots in which *NLP1*, *NLP3*, *NLP4*, or *NLP5* gene is downregulated by using RNA interference (RNAi) developed mature nodules in the presence of a high concentration of NO_3_^−^ (7.5 mM) that normally inhibits the nodule development in wild type plants. On the other hand, transgenic roots overexpressing the *NLP1* gene exhibit hypersensitivity in nodule development in response to a high level of NO_3_^−^. The genetic analysis further shows that *NLP1* and *NLP4* functions are partially redundant in the NO_3_^−^ inhibition of nodule development (Lin et al., 2018). These results indicate an important role of *M. truncatula* NIN-like proteins (MtNLPs) in the NO_3_^−^ inhibition of nodule development.

In *Arabidopsis thaliana*, there are nine NLP proteins all of which have been shown to bind directly to a NO_3_^−^-responsive *cis*-element and regulate the expression of NO_3_^−^-responsive genes (Konishi and Yanagisawa, 2013). In *L. japonicus*, all five NLP proteins, including NIN, can bind to a NO_3_^−^-responsive *cis*-element and regulate downstream gene expression (Soyano et al., 2015). Interestingly, an amino-terminal of NLP proteins is responsible for the NO_3_^−^ activation of NLP proteins. However, unlike other NLP proteins, an N-terminal domain of LjNIN is not responsive to NO_3_^−^. Furthermore, LjNIN antagonizes the NO_3_^−^-induced gene expression in *L. japonicus* (Soyano et al., 2015).

In *A. thaliana*, NLP7 is retained in the nucleus in response to NO_3_^−^ to regulate the expression of early NO_3_^−^-responsive genes (Marchive et al., 2013). In *M. truncatula*, the mostly cytoplasmically localized NLP1 protein, whose gene expression is not induced by NO_3_^−^, accumulates in the nucleus after 10 min of NO_3_^−^ treatments, and this NO_3_^−^ response requires the N-terminal domain of MtNLP1. The NO_3_^−^-induced nuclear retention of NLP proteins appears to be conserved in *A. thaliana* (Marchive et al., 2013), *L. japonicus* (Nishida et al., 2018), and *M. truncatula* (Lin et al., 2018) and requires phosphorylation of the conserved Ser226 in the N-terminal domain of NLP proteins (Liu et al., 2017). MtNLP1ΔN (deletion of the N-terminal domain of MtNLP1) exhibits constitutive nuclear localization (Lin et al., 2018).

MtNIN interacts with all five MtNLPs *via* the PB1 domain (Lin et al., 2018). When co-expressed in *Nicotiana benthamiana* leaves, MtNLP1 suppresses the expression of MtNIN-activated genes, *CRE1* and *NF-YA1* only in the presence of NO_3_^−^ (Lin et al., 2018). However, MtNLP1ΔN (containing the RWP-RK and PB1 domains) inhibits MtNIN-activated *CRE1* and *NF-YA1* expression in the absence of NO_3_^−^. Together with the genetic evidence, these results support the following model: when NO_3_^−^ is below a threshold level, NLP1 and/or other NLP proteins remain largely in the cytosol. Nod factors induce the expression of *NIN*, which in turn activates the expression of downstream genes including *NF-YA1* and *CRE1* that are essential for rhizobial infection and nodule development, respectively. In the presence of a high level of NO_3_^−^, a Nod factor induction of *NIN* is lower, NLP1 and/or other NLP proteins accumulate in the nucleus, where NLP1 suppresses the NIN-activated gene expression by directly binding NIN, competing with NIN for binding sites in the promoters of target genes, or a combination of both, thus inhibiting nodule formation (Lin et al., 2018).

Nitrate greatly inhibits the NIN activation by rhizobia. In the *nlp1* mutant, the NO_3_^−^ inhibition of *NIN* expression in response to rhizobia is less severe, revealing a different mode of action of NLP1 in the NO_3_^−^ regulation of nodulation (Lin et al., 2018). It is known that the NO_3_^−^ inhibition of nodulation can occur locally or systemically *via* a long-range AON pathway (Nishida et al., 2018). Grafting experiments show that the role of MtNLP1 in the NO_3_^−^ regulation of nodulation and lateral root development is determined by roots (Lin et al., 2018).

Recent studies have shown that NLP1 accumulates in the nucleus in response to NO_3_^−^, binds directly to the promoter sequence and activates the expression of *CLE35*, which plays a key role in the long-distance, systemic inhibition of nodule initiation in a SUNN-dependent manner (Luo et al., 2021; Figure 1).

In *L. japonicus*, *NITRATE UNRESPONSIVE SYMBIOSIS1* (*NRSYM1*; *LjNLP4*) is involved in the NO_3_^−^ inhibition of nodule development, *nrsym1* mutant displays strong NO_3_^−^ tolerance such that the number of nodules, infection threads, and nitrogenase activity are not reduced by NO_3_^−^ (Nishida and Suzaki, 2018; Nishida et al., 2018). NRSYM1 accumulates in the nucleus in response to NO_3_^−^ and binds directly to the promoter of *CLE-RS2* and activates its expression in a NO_3_^−^-dependent manner (Nishida et al., 2018; Figure 1). LjNIN can also bind the same *cis*-element in a *CLE-RS2* promoter to activate the *CLE-RS2* expression. Ectopic expression of *LjNIN* interferes with the expression of NO_3_^−^-induced genes, and NO_3_^−^ in turn, downregulates the expression of NIN target genes (Nishida et al., 2018; Figure 1). Genetic, biochemical, and grafting experiments have shown that NRSYM1 accumulated to the nucleus in response to NO_3_^−^ activates *CLE* expression and reduces the number of nodules by an AON pathway (Nishida et al., 2018; Figure 1). Additionally, NRSYM1 is also involved in other nodulation processes, including the infection threads formation and nodule development and function (Nishida et al., 2018).

## The Role Of NO_3_^−^ And NH_4_^+^ Transporters In Symbiotic Nitrogen Fixation

It is known that a low level of external NO_3_^−^ promotes but a high level of NO_3_^−^ inhibits symbiotic N fixation (Streeter and Wong, 1988; Nishida and Suzaki, 2018). The uptake and transport of NO_3_^−^ in plants require two families of proteins, NITRATE TRANSPORTER1/PEPTIDE TRANSPORTER family (NPF; also called NRT1) and NITRATE TRANSPORTER2 (NRT2; Wang et al., 2020a). The larger NPF family consists of 53, 86, and 97 members in *A. thaliana*, *L. japonicus*, and *M. truncatula*, respectively, which are mostly low-affinity NO_3_^−^ transporters with some exceptions such as *Arabidopsis* NRT1.1 and *Medicago* NRT1.3 exhibiting dual-affinity transporter activities (Morere-Le Paven et al., 2011; Wang et al., 2018, 2020b). The NRT2 family consists of only seven, four, and three members in *A. thaliana*, *L. japonicus*, and *M. truncatula*, respectively, and they are high-affinity NO_3_^−^ transporters. Transcriptomic analyses have identified a large number of transporter-encoding genes that are induced in nodules (Colebatch et al., 2004; Takanashi et al., 2012; Laloum et al., 2014). This includes NPF and NRT2 genes (Criscuolo et al., 2012; Clarke et al., 2015) although the role of these proteins in nodule functioning has been reported only for a few of the family members.

Recently, *M. truncatula NPF7.6* has been identified based on its nodule-specific expression pattern (Wang et al., 2020a). *MtNPF7.6* is inducible by rhizobial infection or by a high level of NO_3_^−^, and in nodules, it is specifically expressed in nodule vascular tissues. *MtNPF7.6* is localized to the plasma membrane of nodule transfer cells (NTCs) in the nodule vascular tissues. Experimental evidence support that it mediates the uptake of NO_3_^−^ into nodules. Genetic analysis of *MtNPF7.6* knockout mutants using the CRISPR-Cas9 gene-editing technique shows that *npf7.6* mutants exhibit severely reduced responsiveness to NO_3_^−^ at both low and high levels. Nodules developed on *npf7.6* mutant roots exhibit the accumulation of a high level of NO, a severe reduction of *LEGHEMOGLOBIN* (*Lb*) gene expression, and impairment of nitrogenase activity, supporting a role for NPF7.6-mediated NO_3_^−^ transport in regulating *Lb* gene expression, NO homeostasis, and nitrogenase activity. Because *NPF7.6* is expressed in pericycle, xylem, and phloem tissues in nodules, with NPF7.6 proteins localized on the plasma membrane of NTCs of pericycle, xylem, and phloem, these results suggest that NPF7.6 mediates not only the uptake of NO_3_^−^ from an external environment but also xylem-to-phloem transport of NO_3_^−^, and these processes appear to be critical for the NO_3_^−^ regulation of nodule development and function (Wang et al., 2020a).

In *L. japonicus*, LjNRT2.4, a member of the NRT2 family of high-affinity NO_3_^−^ transporters has been identified to play a role in the positive regulation of symbiotic N fixation by a low, permissible concentration of NO_3_^−^ (Valkov et al., 2020). *LjNRT2.4* is expressed in root and nodule vascular tissues. Comparing the symbiotic and nonsymbiotic growth in the presence of a low NO_3_^−^ concentration (100 μM) of *nrt2.4* mutants and wild type indicates that *nrt2.4* mutants have a mild but significant reduction in the shoot biomass, nodule nitrogenase activity, and nodule NO_3_^−^ content under the symbiotic condition. These phenotypes are even more pronounced when plants are grown in hydroponic culture (hypoxic condition). These phenotypic differences between wild type and *nrt2.4* correlate well with a reduced level of NO in nodules of the *nrt2.4* mutants in *M. truncatula* and *L. japonicus* (Horchani et al., 2011; Valkov et al., 2020). These studies support the role of NO_3_^−^-NO respiratory cycle in both mitochondria and bacteroids of nodule-infected cells as an alternative pathway for the energy production for symbiotic N fixation, which is particularly important under hypoxic conditions (Horchani et al., 2011; Valkov et al., 2020).

Ammonium levels in soils are usually 10–1,000 times less than that of NO_3_^−^, but some plants have preferentially taken up NH_4_^+^ when both NO_3_^−^ and NH_4_^+^ are present (Gazzarrini et al., 1999). At a high level, NH_4_^+^ inhibits symbiotic N fixation although the regulatory mechanisms appear to be different from those of NO_3_^−^ (Lin et al., 2018). There are two families of related transporter proteins, AMMONIUM TRANSPORTER1 and 2 (AMT1 and AMT2) that mediate NH_4_^+^ uptake from the soil and transport within the plants. In *L. japonicus*, *AMT1;1*, *AMT1;2*, and *AMT1;3* can complement a yeast NH_4_^+^ transporter mutant and encoded proteins exhibit a high-affinity NH_4_^+^ transporter activity (D’Apuzzo et al., 2004). The expression of these three genes exhibits broad tissue specificity (D’Apuzzo et al., 2004). In nodules, the expression of *LjAMT1;1* and *LjAMT1;2* is in outer cortical cells and peripheral tissues, and the infected cells and vasculature tissues of the nodule, respectively (D’Apuzzo et al., 2004; Rogato et al., 2008). Furthermore, the expression of *LjAMT1;1* and *LjAMT1;2* is not responsive to NH_4_^+^ (Rogato et al., 2010). The expression of *LjAMT1;3*, however, is induced by NH_4_^+^ in a dose-dependent manner (Rogato et al., 2010). Knockdown of *LjAMT1;1* expression using RNAi with a nodule-infected cell-specific *Lbs* promoter results in transgenic plants developing nodules with a compromised nitrogenase activity but a significantly increased number of nodules, supporting a role for *LjAMT1;1*-mediated NH_4_^+^ transport in nodule development and functioning (Rogato et al., 2008). In *M. truncatula*, three *AMT2* family members, *AMT2;3*, *AMT2;4*, and *AMT2;5*, are induced in roots during symbiosis with arbuscular mycorrhizal fungi. Genetic analyses show that *MtAMT2;3* but not *MtAMT2;4* is required for the suppression of premature arbuscule degeneration (PAD) in *phosphate transporter4* (*pt4*) mutants, supporting a role of the N status mediated by *AMT2;3* in controlling arbuscule life span (Breuillin-Sessoms et al., 2015).

## The Involvement of NO in Symbiotic Nitrogen Fixation

Nitric oxide, a reactive N species (RNS), is ubiquitous in plants and plays a signaling role in diverse developmental processes, including different stages during legume–rhizobial symbiosis. Both legumes and rhizobial partners contribute to the production and scavenging of NO during symbiotic N fixation. Enzymes, including NITRATE REDUCTASE (NR), PLASMA MEMBRANE-BOUND NITRITE: NO REDUCTASE, mitochondrial-electron transport chain (mETC)-dependent nitrite-reducing activity, NO synthase (NOS), POLYAMINE OXIDASE and HYDROXYLAMINE OXIDASE, and nonenzymatic reduction of nitrite have been suggested to contribute to NO production (Stohr et al., 2001; Bethke et al., 2004; Planchet et al., 2005; Tun et al., 2006; Corpas et al., 2009; Rumer et al., 2009; Horchani et al., 2011; Meilhoc et al., 2011; Blanquet et al., 2015; Astier et al., 2018; Leon and Costa-Broseta, 2020). NO production is enhanced by environmental stresses such as hypoxia and NO_3_^−^, and NO has been suggested to play a role in the NO_3_^−^ inhibition of the nitrogenase activity (Kato et al., 2010).

Nitric oxide has been reported to both promote and inhibit symbiotic N fixation. These contradictory effects of NO may be associated with the level and pattern of NO production during legume–rhizobia interaction and nodule development (Signorelli et al., 2020). During symbiotic interactions between *L. japonicus*-*Mesorhizobium loti* and *M. truncatula*-*Sinorhizobium meliloti*, NO was induced at 4-h postinoculation (hpi; Nagata et al., 2008). Studies have shown that NO was induced in the infection pocket and specific scavenging of NO resulted in delayed development of nodules (del Giudice et al., 2011). Transcriptomic analyses revealed that NO can suppress defense responses, which would promote rhizobial infection and nodule development (Ferrarini et al., 2008; Boscari et al., 2013). Treatments with a low-level NO donor sodium nitroprusside (SNP, 0.1 mM) promoted the nitrogenase activity of nodules in *L. japonicus* although the mechanisms were not clear (Kato et al., 2010). These observations support a positive role of NO in the legume–rhizobia interaction and nodule development.

Treatments with a higher concentration of SNP (1 mM) inhibited the nitrogenase activity of nodules in *L. japonicus* (Kato et al., 2010). A similar situation was previously reported in treatments *in vitro* of nitrogenase isolated from the bacteroids of soybean (Trinchant and Rigaud, 1982). These observations support that NO can directly interact with and inhibit the activity of nitrogenase in soybean (Trinchant and Rigaud, 1982).

Nitric oxide can modulate the activity of proteins through S-nitrosylation, a reversible modification of a protein in which NO covalently binds to the thiol group of a cysteine residue of the protein to form an S-nitrosothiol. The modification results in structural changes and thereby may affect the activity of the protein. It is known that NO inhibits the activity of nitrogenase *via* the formation of a metal-nitrosyl complex (Michalski and Nicholas, 1987) or S-nitrosylation (Puppo et al., 2013). In addition to nitrogenase, many proteins related to the tricarboxylic acid (TCA) cycle and carbohydrate metabolism, amino acid metabolisms, such as ASPARAGINE SYNTHETASE, GLUTAMINE SYNTHETASE, S-ADENOSYLMETHIONINE SYNTHETASE, and redox regulation GLUTATHIONE PEROXIDASE1 are S-nitrosylated and inactivated by NO during symbiosis (Melo et al., 2011; Puppo et al., 2013; Castella et al., 2017).

LEGHEMOGLOBIN proteins transport and deliver a low but steady level of O_2_ to symbiosomes for bacteroid respiration, which is required for symbiotic N fixation but without inactivating the nitrogenase enzyme by O_2_. In addition to *Lbs*, plants also synthesize HEMOGLOBINs (Hbs), called Phytoglobins or Phytogbs, which can be divided into six groups based on the sequence and biochemical properties such as affinities to O_2_ (Hill et al., 2016). In *L. japonicus* nodules, all types of Phytogbs are highly expressed (Bustos-Sanmamed et al., 2011). At present, the function of Phytogbs remains to be investigated in detail. Under the microaerobic condition in nodules, NO produced by mETC is diffused to the cytosol and is oxidized to NO_3_^−^ by OXYPHYTOGLOBIN [Phytogb(Fe^2+^)O_2_] and subsequently NO_3_^−^ is reduced back to nitrite by NR and transported to mitochondria, and this cycle is repeated. This Phytogb-NO cycle is required for the re-oxidation of NAD(P)H to NAD(P)^+^, which is used in the glycolytic process (Igamberdiev and Hill, 2004).

In the N fixation zone of nodules, the NO level is high (Puppo et al., 2013). Both *Lbs* and Phytogbs contributed to the scavenging of NO and O_2_, and this is important for preventing the inactivation of the nitrogenase enzyme by NO (Herold and Puppo, 2005; Puppo et al., 2013; Berger et al., 2019). In *S. meliloti*, a FLAVOHEMOGLOBIN (HMP) has been shown to play a key role in detoxifying NO (Meilhoc et al., 2010; Cam et al., 2012).

## The Role of Phosphate Signaling and Transport in Symbiotic Nitrogen Fixation

Phosphate starvation response (PSR) includes both a long-distance, systemic response that involves sensing of cellular Pi level and a local, root tip inhibition response that involves sensing of extracellular Pi availability (Abel, 2017; Puga et al., 2017; Ried et al., 2021). Cellular Pi sensing relies on the MYB coiled-coil (MYB-CC) transcription factors, PHOSPHATE STARVATION RESPONSE1 (PHR1) and its related proteins PHLs (Rubio et al., 2001) and inositol pyrophosphates (PP-insPs)-binding SPX proteins that exhibit Pi-dependent inhibition of PHR activities (Puga et al., 2014; Wild et al., 2016; Ried et al., 2021). Under conditions of sufficient Pi, SPX binds to the coiled-coil domain of PHR and prevents the transcription factor from binding the *cis*-element in the promoter sequences of *PSI* genes and from activating their expression (Puga et al., 2014; Wang et al., 2014; Wild et al., 2016). Under the condition of Pi deficiency, PHR1 directly induces the transcription of *PHT1* and *PHOSPHATE TRANSPORTER TRAFFIC FACILITATOR1* (*PHF1*), encoding an endoplasmic reticulum (ER) exit cofactor that interacts with and promotes PHT1 traffic to the plasma membrane (Gonzalez et al., 2005; Bayle et al., 2011; Chen et al., 2011).

Phosphate acquisition and transport are the key processes for efficient symbiotic N fixation in legumes (Nussaume et al., 2011; Gu et al., 2015; Sulieman and Tran, 2015). In a soybean, GmPT5, a high-affinity Pi transporter has been shown to transport Pi from roots to nodules, especially under conditions of limited P (Qin et al., 2012). Interestingly, another nodule-localized Pi transporter GmPT7 appears to play a role in delivering Pi from an external environment to the nodule fixation zone (Chen et al., 2019). PHR1, the constitutively expressed MYB-domain transcription factor, and its downstream target PHT1 constitute the key regulatory modules for symbiotic N fixation in legumes (Nussaume et al., 2011; Lu et al., 2020). Added to its complexity, it has been shown that a single soybean GmPHR has more than one GmPHT1 target, and one GmPHT1 is under the control of multiple GmPHRs (Lu et al., 2020). Therefore, different combinations of Pi transporters and Pi stress response factors form diverse regulatory networks in the N fixation and non-fixation zones to maintain Pi homeostasis in response to both systemic and local Pi availability for efficient symbiotic N fixation in soybean nodules (Lu et al., 2020). Eleven members of the PT1 family have been identified in *M. truncatula*, and most of them were highly expressed under conditions of low Pi (Cao et al., 2020). In addition to root epidermis, cortex, vascular tissues, and root tips, *MtPT6* was also expressed in nodules, suggesting that it may play a role in Pi uptake from soil and transporting Pi to nodules from other tissues (Cao et al., 2020). Pi deficiency induces the expression of purple acid phosphatase (PAPs) in root nodules, which leads to an enhanced acquisition and utilization of P in root nodules (Li et al., 2017; Wang et al., 2020e). Furthermore, under conditions of Pi starvation, GmPHR1 can bind to the promoter sequence and activate the expression of *GmPAP12* to maintain the P homeostasis and N fixation (Wang et al., 2020e).

**Figure 2:**
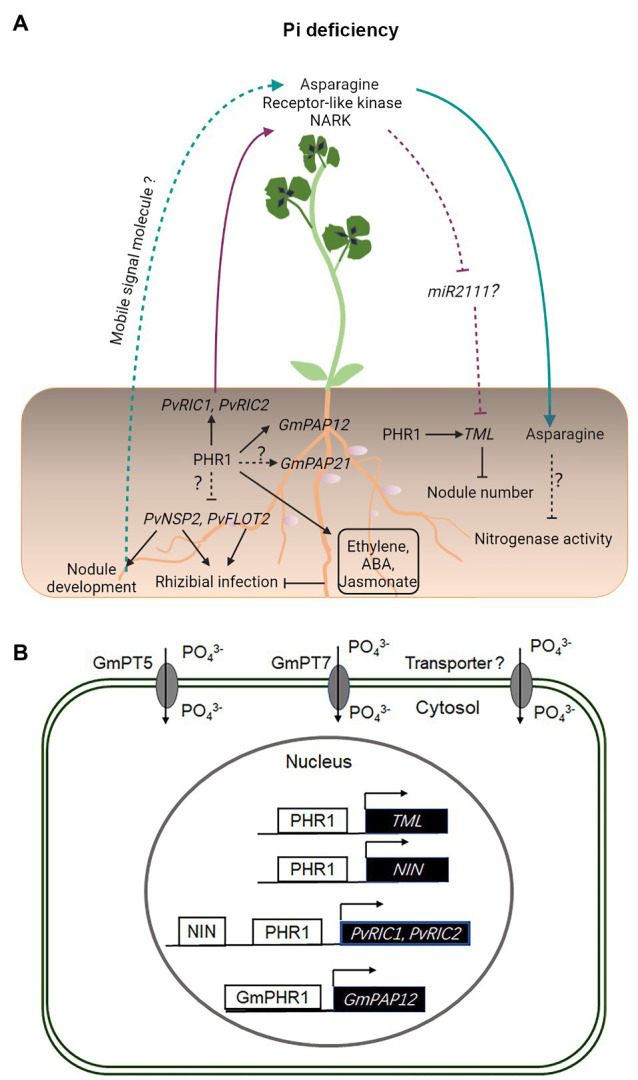
Legume–rhizobium symbiotic signaling under inorganic phosphate (Pi) deficiency. Under Pi deficiency, the expression of *PvNSP2* and *PvFLOT2* is inhibited, resulting in compromised rhizobial infection and nodule development **(A)**. Pi deficiency induces the expression of *GmPAP21* and *GmPAP12*, the latter of which is directly regulated by GmPHR1 **(A,B)**. Pi deficiency also activates ethylene, abscisic acid (ABA), and jasmonate-related signaling processes, which inhibit rhizobial infection in *Phaseolus vulgaris* **(A)**. The expression of *NIN*, *RIC1*, *RIC2*, and *TML* is directly activated by PHR1 in *P. vulgaris* under conditions of Pi deficiency **(A,B)**. PvRIC1 and PvRIC2, two CLE-like peptides, negatively regulate the nodule number *via* the LRR-RLK, NARK-dependent AON pathway **(A)**. It has not been shown whether *miR2111* is involved in this process **(A)**. Under Pi deficiency, the N-compound asparagine is significantly increased in the phloem sap and has been suggested to act as a systemic signal to inhibit the nitrogenase activity in nodules **(A)**. Under Pi deficiency, GmPT5 and GmPT7 are activated and function to mobilize Pi from internal and external sources to nodules **(B)**. Solid and dashed lines indicate the processes that are supported by experimental evidence and putative, respectively.

Phosphate deficiency negatively impacts early molecular and physiological responses to rhizobial infection in common bean (*Phaseolus vulgaris*), including a significantly delayed activation of *PvNSP2* and *PvFLOT2* genes, compromised the root hair deformation and infection thread development, and the formation of a reduced number of nodules (Isidra-Arellano et al., 2018; Figure 2). These defects may be associated with an auxin-cytokinin imbalance, upregulation of ethylene, abscisic acid (ABA), and jasmonate-related signaling pathways under Pi deficiency in common bean (Isidra-Arellano et al., 2018). Recent studies have shown that the AON-related genes *PvNIN*, *PvRIC1*, *PvRIC2*, and *PvTML* are induced under conditions of Pi deficiency and their transcriptional activation is likely to be dependent on PHR1 (Isidra-Arellano et al., 2020; Figure 2). These studies suggest that Pi deficiency inhibits the root nodule symbiosis in common bean and soybean through constitutive activation of the AON pathway under Pi deficiency (Isidra-Arellano et al., 2020).

Phosphate deficiency leads to a decreased nodulation and nitrogenase activity in many legume species analyzed so far. Although the underlying molecular mechanisms remain largely unknown, studies have shown that Pi deficiency resulted in changes in the composition of free amino acids, particularly a significant increase of asparagine in the phloem sap, supporting the notion that the shoot-derived asparagine acting as a systemic signal to translocate to nodules and inhibit the nitrogenase activity in white clover (*Trifolium repens*) and *M. truncatula* (Almeida et al., 2000; Sulieman et al., 2013). Similarly, NO_3_^−^ treatments also significantly increased the production of the N-compound asparagine in the shoot of nodulated soybean plants (Bacanamwo and Harper, 1997), supporting that the inhibition of nodulation by both Pi starvation and NO_3_^−^ application may be due to an N-feedback regulation (Bacanamwo and Harper, 1997; Almeida et al., 2000; Sulieman et al., 2013).

Earlier studies have suggested that increased permeability of nodules to oxygen may explain the Pi deficiency-induced inhibition of the nodule function in common bean (Hernandez and Drevon, 1991; Lazali and Drevon, 2014). Physiological studies of different legumes have revealed that Pi is preferentially relocated from other organs to nodules under Pi deficiency (Sa and Israel, 1991; Tang et al., 2001). It has been shown that a high phytase activity in nodules constitutes a mechanism for faba bean (*Vicia faba* L.)-rhizobia symbiosis to adapt symbiotic N fixation to conditions of Pi deficiency (Makoudi et al., 2018). In white lupin (*Lupinus albus*), Pi deficiency results in enhanced nodulation in cluster root zones and an increased potential for organic acid production in root nodules, contributing to the resilience of white lupin to P-deficiency (Schulze et al., 2006).

**Figure 3:**
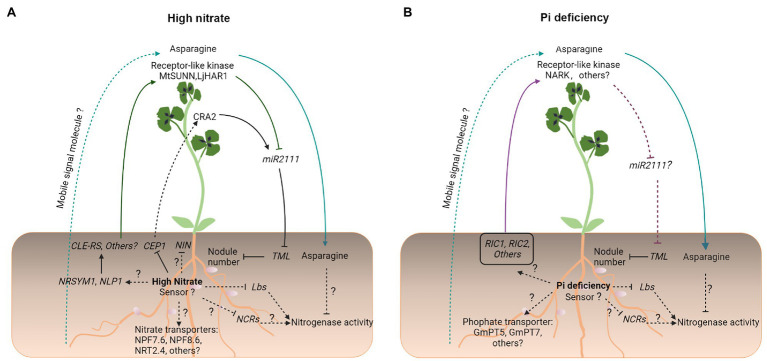
An overview of symbiotic signaling under high NO_3_^−^ and Pi deficiency in legumes. Under conditions of **(A)** high NO_3_^−^ and **(B)** Pi deficiency, compromised legume–rhizobial symbiotic interactions share both similar signaling processes and unique processes. Similar symbiotic signaling processes include enhanced CLE-like/LRR-RLK-dependent AON signaling, a significant increase in the production and transport of the N-compound asparagine from the shoot to root nodules, where it inhibits nitrogenase activity, and reduced the expression of genes encoding *Lbs* and NCR peptides, which are essential to maintain the nitrogenase activity. Unique symbiotic signaling processes include transport, initial signal perception, and transduction. Solid and dashed lines indicate the processes that are supported by experimental evidence and putative, respectively. Question marks indicate unknown pathways.

A comprehensive transcriptome profiling analysis has shown that NO_3_^−^ application and Pi starvation both reduce symbiotic N fixation *via* similar changes in the nodule gene expression in *M. truncatula* (Liese et al., 2017). Particularly, in both treatments, the activity of nitrogenase was targeted by a reduction in the expression of nodule cysteine-rich (*NCR*) genes (encoding nodule-specific cysteine-rich peptides), by a disturbance of iron allocation in nodule inner cells, and by a strong reduction in the expression of *Lbs*, which may restrict the supply of oxygen for respiration (Liese et al., 2017; Figures 2, 3).

## Transcriptional Regulation of Phosphate Starvation and Nitrate Responses

Under Pi starvation, plants upregulate Pi uptake but downregulate NO_3_^−^ uptake (Wang et al., 2020c). An MYB-related GARP-type transcription factor NITRATE-INDUCIBLE, GARP-TYPE TRANSCRIPTIONAL REPRESSOR1.2 (NIGT1.2) has been identified to bind specific promoter sequences of *PHT1;1* and *PHT1;4*, and also *NRT1.1* in *A. thaliana* (Wang et al., 2020c). *NIGT1.2* is induced by Pi-starvation and NO_3_^−^ treatments (Wang et al., 2020b,c). Overexpression of *NIGT1.2* results in an enhanced Pi uptake capacity and Pi levels in transgenic plants. On the contrary, *nigt1.1 nigt1.2* double mutants exhibit a hypersensitive phenotype and significantly reduced Pi uptake capacity and levels under conditions of Pi deficiency although *nigt1.2* single mutants were similar to wild type plants under the growth conditions of Pi sufficiency and Pi deficiency (Wang et al., 2020c). These results support a positive role of NIGT1.2 (also NIGT1.1) in Pi uptake under Pi-limitation.

Interestingly, Pi-limitation reduces the NO_3_^−^ uptake and accumulation. The overexpression of *NIGT1.2* results in a reduced NO_3_^−^ influx and accumulation in transgenic plants. On the other hand, the *nigt1.1 nigt1.2* double mutant exhibits a significantly increased NO_3_^−^ influx only under Pi-limitation (Wang et al., 2020c). These results support a negative role of NIGT1.2 (also NIGT1.1) in the uptake of NO_3_^−^ under Pi-starvation. NIGT1.2 binds AGANNNAAA and AAACNNAACC *cis*-elements in the promoter sequences of *PHT1;1* and *PHT1;4* both *in vitro* and *in vivo*, supporting a direct, transcriptional upregulation of *PHT1;1* and *PHT1;4* by NIGT1.2 under Pi-limitation (Medici et al., 2015; Wang et al., 2020c). Intriguingly, NIGT1.2 also binds AANNAGA, TGGGA, and GAGA *cis*-elements in the *NRT1.1* promoter sequence, and downregulates the expression of *NRT1.1* under Pi-starvation (Medici et al., 2015; Wang et al., 2020c).

## Conclusion and Future Perspectives

Much progress has been made in our understanding of N and P signaling and transport during legume–rhizobium symbiosis (Figure 3). It is now known that the NLP family of transcription factors plays a key role in the inhibitory effect on legume–rhizobium symbiosis of a high level of NO_3_^−^. A high level of NO_3_^−^ causes the retention and accumulation of NLP proteins in the nucleus, which inhibit the transcriptional activity of NIN in activating the expression of Nod factor-inducible genes such as *CRE1* and *NF-YA1* (Lin et al., 2018; Nishida et al., 2018; Figure 1). NLPs can interact directly with NIN, bind the same *cis*-elements as NIN, or both (Lin et al., 2018). Unlike those of NLPs, the N-terminal of NIN is not responsive to NO_3_^−^ and the function of NIN also diverges from that of NLPs (Suzuki et al., 2013). These changes are thought to play an important role in the evolution of legume–rhizobium symbiosis. On the other hand, a high level of NO_3_^−^ reduces the level of Nod factor-induced *NIN* expression (Barbulova et al., 2007). However, the mechanisms remain elusive.

A low level of NO_3_^−^ promotes nodule development and symbiotic N fixation. However, the regulatory mechanisms remain largely unknown. In plants, there are two families of transporters for NO_3_^−^ transport, a large NPF/NRT1 family of mostly low-affinity transporters (>80 members in *L. japonicus* and *M. truncatula*), and a small NRT2 family of high-affinity NO_3_^−^ transporters (<5 in *L. japonicus* and *M. truncatula*; Criscuolo et al., 2012; Sol et al., 2019). The high-affinity LjNRT2.4 protein expressed in root and nodule vascular tissues has been identified to play a positive role in the regulation of nodule development and function by a low level of NO_3_^−^, supporting a NO_3_^−^–NO cycle being an alternative energy source for mitochondria and bacteroids and this is particularly important for symbiotic N fixation under a hypoxia condition (Valkov et al., 2020). The involvement in nodule development and function has been demonstrated only for a limited number of NO_3_^−^ transporters. The presence of a large number of NO_3_^−^ transporters supports the importance and complexities of these groups of proteins in regulating uptake and transport of NO_3_^−^ during plant and/or nodule development and function and this needs to be demonstrated in future investigation.

Inorganic Pi signaling and transport have been investigated extensively in model organisms such as *A. thaliana* and rice. PSR appears to involve both a long-distance, systemic response that senses cellular Pi levels, and a local, root tip inhibition response that senses extracellular Pi availability (Wang et al., 2020d). Sensing of cellular Pi levels relies on the PHR1-PHT1 module (Puga et al., 2017; Lu et al., 2020), which is a multicomponent, transcriptional, and posttranscriptional regulatory network. The local, root tip inhibition response to Pi starvation includes both a rapid reduction of cell elongation at the root transition zone and a gradual inhibition of cell division at the root apical meristem (RAM) and is tightly linked to the accumulation of Fe^3+^ in the apoplast, production of ROS, and formation of callose at RAM and transition zone, which require LPR1 (multicopper oxidase), PDR2 (P5-type ATPase), ALMT1 (malate transporter), and STOP1 (zinc finger transcription factor; Ticconi et al., 2009; Muller et al., 2015; Balzergue et al., 2017; Naumann et al., 2019). The local response to Pi levels shares some components involved in aluminum toxicity response and DNA damage response, supporting a critical role of a sufficient level of Pi in maintaining the basic function of root tip cells.

Interestingly, there is an antagonistic interaction between Pi and NO_3_^−^. Under Pi- starvation, plants tend to upregulate Pi transport but downregulate NO_3_^−^ transport. A GARP-type transcription factor, NIGT1.2 (also NIGT1.1) appears to directly upregulate the expression *PHT1;1* and *PHT1;4* genes and downregulate *NRT1.1* expression through binding of different *cis*-elements in their promoter sequences (Wang et al., 2020c). Although much progress has been made in the field of N and P signaling and transport regulation in plant growth and development, only a limited number of transporter-encoding genes have been investigated during legume–rhizobium symbiosis (Figure 3). In addition to N and P, efficient symbiotic N fixation also requires sufficient supplies of other mineral elements, such as potassium, sulfur, calcium, magnesium, iron, and zinc (Garcia et al., 2020). With the development of some genetic resources in different legume species, including *L. japonicus*, *M. truncatula*, and *Glycine max*, and CRISPR/Cas9 gene-editing tools (Wolabu et al., 2020; Zhu et al., 2021), it is foreseeable that a better understanding of signaling, transport, and interactions of these mineral elements during nodule development will make it possible to improve the efficiency of symbiotic N fixation of legume crops to contribute to sustainable agriculture.

## Author Contributions

RC and YM conceived the project and wrote the manuscript. YM prepared the figures. All authors contributed to the article and approved the submitted version.

### Conflict of Interest

The authors declare that the research was conducted in the absence of any commercial or financial relationships that could be construed as a potential conflict of interest.
